# Indoor Magnetic Signature Based Localization Algorithm without Person-Dependent Parameter Calibration

**DOI:** 10.3390/s140814375

**Published:** 2014-08-07

**Authors:** Young Soo Suh, Baatardorj Amarbayasgalan

**Affiliations:** Department of Electrical Engineering, University of Ulsan, Namgu 680-749, Ulsan, Korea; E-Mail: ashoi amu@yahoo.com

**Keywords:** inertial sensor, magnetic sensor, magnetic signature, magnetic disturbance, localization

## Abstract

Location-dependent differences of ambient magnetic fields inside a building can be used to estimate location. In this paper, an inertial/magnetic sensor is attached to a belt position and its location is estimated using the ambient magnetic field. The walking distance is estimated using the linear relationship between the walking step length and the maximum acceleration during the step. The magnetic field data during walking is compared with a pre-collected magnetic signature. In this process, calibration steps are required for two person-dependent parameters : the walking step length estimation parameter and the hard iron parameter. An adaptive algorithm is proposed, in which these person-dependent parameters are estimated in addition to the location. Thus no person-dependent parameter calibration process is required. Through experiments, it is shown that the location and parameters are estimated accurately.

## 1. Introduction

Recently much attention has been paid to indoor navigation [1], where a person's location is estimated in an indoor environment. The main applications of an indoor navigation algorithm are for first responders [2], handicapped people [3] and entertainment [4].

Techniques for indoor navigation can be divided into two groups. One is an inertial sensor-based method, which provides relative position by estimating heading and walking distance [5–8]. The main advantage of this method is that it does not require any infrastructure in the environment or prior knowledge of it. The main disadvantage is that only the relative position is provided and its position error becomes larger over time.

The other techniques are methods that require infrastructure in the building or prior knowledge of it. In [9], wireless signals are used to estimate the absolute location in the building. In [3,10,11], location-to-location variation of the magnetic field has been used to estimate position. The main advantage of this method is that it provides the absolute position and its position error is bounded. The main disadvantage is that it requires full knowledge of the indoor environment and it could be sensitive to wireless or magnetic disturbances.

Many practical navigation algorithms have tried to combine inertial sensor-based navigation algorithms with an absolute positioning method to compensate for each method's weakness [11,12]. In the combined algorithms, the inertial navigation algorithm is usually the primary navigation algorithm. The error accumulation of the inertial navigation algorithm is prevented by using an absolute positioning method. Compared with an absolute positioning only method, it requires less thorough knowledge of the indoor environment: if a person is in areas with no knowledge of the environment, the location can still be estimated using the inertial navigation algorithm.

Inertial sensor-based algorithms can be classified into two groups: one is using the inertial navigation algorithm to accurately estimate the position using an inertial sensor unit attached on a shoe [6]. The movement of the foot is estimated using an inertial navigation algorithm providing the three dimensional location. The other group roughly estimates walking length based on the output of accelerometer [8]. The accelerometer output can be used to detect how many steps a person has walked from the fact that one step walking length is related to one step walking time and the maximum acceleration (due to shock when a foot touches the ground). For example, it is known that one step walking length is proportional to the maximum acceleration during the walking step.

Although the first algorithm provides much more accurate position estimation, we used the latter algorithm since the inertial sensor location is not limited to the shoe. We note that inertial sensors should be placed on a shoe in the first algorithm to take advantage of zero velocity updating [13], where the velocity error is compensated using the fact that the velocity is zero when the foot is on the ground. Also, a cheaper accelerometer can be used in the latter algorithm.

Due to iron and other material, magnetic fields inside a building are not the same in different locations [14]. We measure and record magnetic field inside a building (this data is called magnetic signature data). When a person is walking inside a building, the measured magnetic field during walking (using three axis magnetic sensor) can be compared with the magnetic signature data to estimate the location. There are many algorithms that use this magnetic signature. They differ in regard to whether one-point data or multiple-point data are used, and whether the norm or the direct value of the sensor is used.

In [10], a one-dimensional location estimation problem has been considered both for a mobile robot and people. One-point magnetic sensor data was used in the measurement equation of a particle filter, where both the direct sensor value and norm value are tested. It was shown that the direct sensor value gives a better result. In [11], two dimensional location estimation problem was considered for people. A particle filter and one-point magnetic sensor data was used. In [3], multiple-point norms of magnetic sensor values are used to estimate a person's one-dimensional location. Magnetic signature data was collected at certain distance intervals. Magnetic sensor data during walking has also been collected at certain time intervals, where distance information is not known perfectly. This distance uncertainty was resolved using the dynamic warping method [15].

In previous studies, two parameters had to be obtained for each person. One parameter is a magnetic-related parameter due to magnetic elements in clothing, belt, and other items such as keys. This parameter is different for each person. Usually, to identify this magnetic parameter, a procedure of moving a magnetic sensor in a certain pattern is required (for example, a figure eight pattern) [16,17]. When a sensor is attached to the body, it is not always easy to move in a predetermined pattern. The other parameter is related to inertial sensor values and walking distance. Since walking pattern and style are different for each person, this parameter is also person-dependent [18]. To identify this walking parameter, a person needs to walk some known length (for example, using the method in [19]).

If the magnetic parameter is unknown, it is not easy to apply existing methods ([3,10,11]) for location estimation since the measured magnetic sensor data is corrupted by person-dependent magnetic elements. Thus the estimation accuracy using the magnetic signature becomes poor.

If the walking parameter is unknown, the walking distance is inaccurately estimated. Walking distance estimation is important because it is necessary for comparison between magnetic signature data (in which the distance between sampling points is exactly known) and the measured magnetic data during walking (in which the distance between the sampling points is estimated based on the walking distance estimation). Additionally, the location must be estimated using the walking distance when the magnetic signature is not available.

We propose an improved location estimation algorithm using the magnetic signature and assuming that we have the magnetic signature information about a building. The main merit of the proposed method is that it does not require any person-dependent calibration procedure. The two parameters are identified by the algorithm automatically, making the person-dependent calibration unnecessary. To the best of our knowledge, there has been no attempt to identify both parameters using magnetic signature information. The identification of the walking parameter is related to dynamic time warping techniques in [15], where walking distance is estimated. We note that walking parameter identification is not considered in [15].

## 2. Problem Formulation

**Figure 1.**
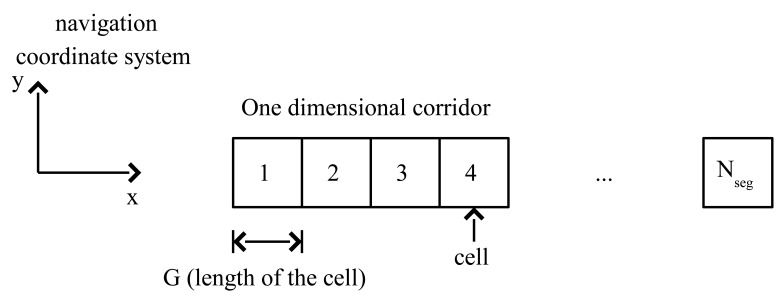
One-dimensional corridor is divided into cells.

The location estimation problem in a one-dimensional corridor is considered (see Figure 1). The corridor is divided into *N_cell_* cells, where *G* is the length of one cell. The location is represented by the cell number *m*.

Two coordinate frames are considered: the navigation coordinate system and the body coordinate system. The *z* axis of the navigation coordinate system is assumed to coincide with the local gravitation direction (the *z* axis is in the upward direction). The *x* axis of the navigation coordinate system coincides with the right direction of the corridor (see Figure 1). The 3 axes of the body coordinate system coincide with the 3 axes of the sensor unit.

**Figure 2.**
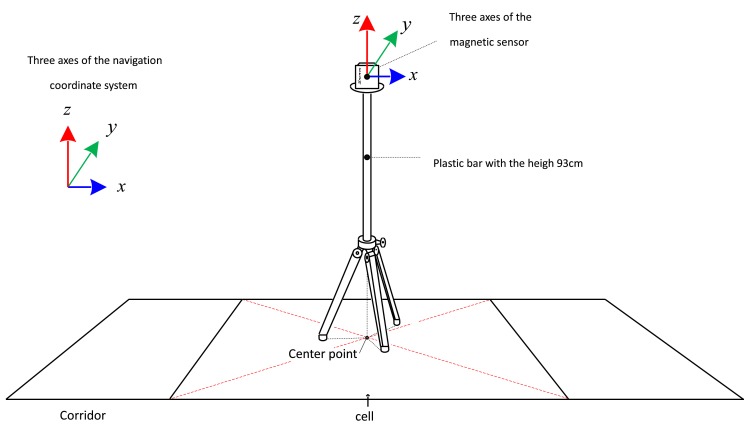
Magnetic signature measurement for each cell.

The magnetic field data at the center of each cell is measured (see Figure 2). The magnetic sensor is located on top of a 93 cm plastic bar (to avoid magnetic disturbance from the bar) and 10 s data (100 samplings per second) are recorded. The average value is used as the magnetic signature for the cell. The symbol *z_sig,m_* ∈ *R*^3^ denotes the magnetic signature of the *m*-th cell. The three axes of the sensor during signature data measurement coincide with the three axes of the navigation coordinate system (see Figure 2). Thus *z_sig,m_* is the magnetic field of the *m*-th cell expressed in the navigation coordinate system.

**Figure 3.**
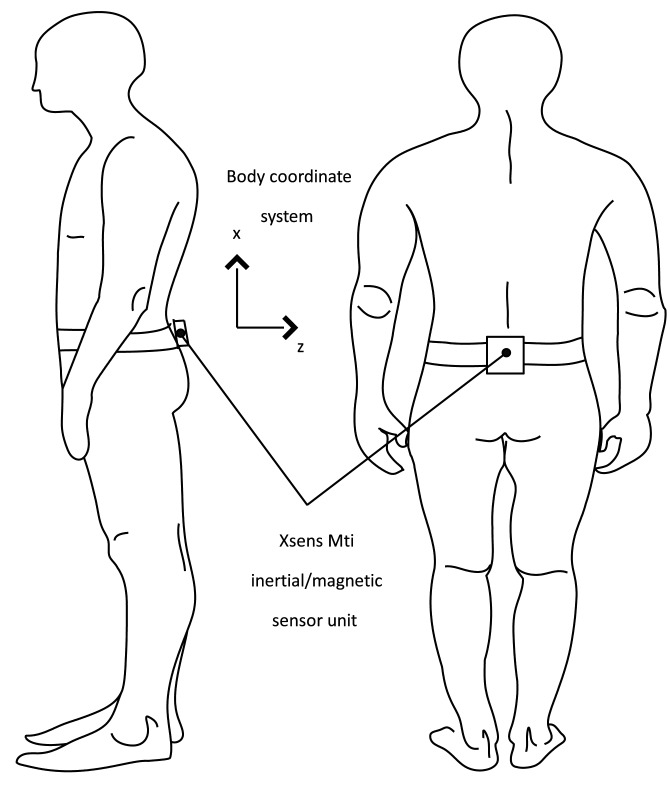
Inertial/magnetic sensor unit on the belt of a person.

The magnetic signature data is compared with the magnetic data during walking to estimate the location. A person carries an inertial/magnetic sensor unit XSens MTi sensor; on the belt (see Figure 3). The belt location is chosen since the attitude of the sensor unit is almost constant during walking. In the proposed method, the attitude change must be small, or else our modeling (1) in Section 3 would not be valid. Thus a sensor unit could be attached on a belt or the chest. The proposed algorithm cannot be used for a sensor unit on a cell phone since its attitude change during walking is large if a person is holding the phone.

The sensor unit consists of 3 axis accelerometers, 3 axis gyroscopes and 3 axis magnetic sensors. The accelerometers are used to estimate walking step length and the magnetic sensors are used to estimate heading (using the earth's magnetic field) and specific location (using the location-specific magnetic signature). Gyroscopes are not used since the proposed algorithm does not require them. If an attitude/heading estimation algorithm or an inertial navigation algorithm were combined with the proposed algorithm, a gyroscope could contribute to enhance the accuracy.

## 3. Segment-Based Location Estimation Using Magnetic Signature Data

Suppose a person is standing at the center of the *m*-th cell and let *y_m_* be the measured magnetic data from the sensor attached to the body. In general, the measured value *y_m_* is different from the magnetic signature data *z_sig,m_* although they are measured at the same location.

The relationship between *y_m_* and *z_sig,m_* can be approximated by the following equation:

(1) $$y_{m}=Cz_{\text{sig},m}+h$$

where *C* ∈ *R*^3×3^ is the rotation matrix and *h* ∈ *R*^3^ represents the hard iron effect due to magnetized iron (such as steel parts in clothing or keys) or a magnetic source (such as a cell phone or a computer). The vector *h* is a person-dependent parameter since clothes and carried items are different for each person. When *h* = 0, *y_m_* is the magnetic field of the *m*-th cell expressed in the body coordinate system (recall that *z_sig,m_* is the same vector expressed in the navigation coordinate system). Thus rotation matrix *C* represents the rotation between the navigation coordinate system and the body coordinate system.

In existing studies [3,10,11], it is assumed that *h* is known in advance through the calibration process. Once *h* is known, we have the following from the fact that *C* is an orthogonal matrix:

(2) $${\left\|y_{m}-h\right\|}_{2}={\left\|z_{\text{sig},m}\right\|}_{2}$$

Equation (2) is used to compare *y_m_* and *z_sig,m_* in existing studies.

An algorithm, which does not require prior knowledge of *h*, is proposed. Since it is difficult to estimate the location using only one magnetic data, a number of magnetic data are grouped into a segment, which is compared with the magnetic signature data. Suppose a person walked a distance (*N_seg_* − 1)*G*, where *N_seg_* is a positive integer. Let *y_seg,i_* ∈ *R*^3^(1 ≤ *i* ≤ *N_seg_*) be the magnetic data obtained during walking, where *y_seg,i_* is the magnetic sensor data obtained when the walking length is (*i* – 1)*G*. Thus *y_seg,i_* is magnetic data obtained at equally spaced locations along the walking path. We call {*y_seg,i_*} a magnetic data segment during walking. The way to obtain this segment is explained in Section 4. Here we just note that the segment value depends on a person-dependent walking-related parameter *α* and thus *y_seg,i_*(*α*) notation is used to emphasize the dependency on *α*. Also note that *y_seg,_*_1_ is the starting point. If a person is walking to the right direction, *y_seg,_*_1_ is the magnetic data obtained at the leftmost position of the segment. If a person is walking to the left direction, *y_seg,_*_1_ is the magnetic data obtained at the rightmost position of the segment.

**Figure 4.**
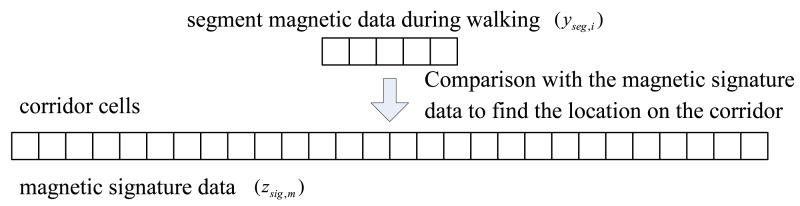
Segment-based magnetic signature comparison.

We want to know the location of this segment on the corridor cells (see Figure 4).

For this purpose, we introduce the performance index *f*(*m, α*, direction):

(3) $$f\left(m,\alpha ,\text{direction}\right)=\{\begin{array}{ll} \min_{C,h\in R^{3}}\sum_{i=1}^{N_{\text{seg}}}w_{i}{\left\|y_{\text{seg},i}\left(\alpha \right)-Cz_{\text{sig},m+i-1}-h\right\|}_{2}^{2} & \text{right direction}\\ \min_{C,h\in R^{3}}\sum_{i=1}^{N_{\text{seg}}}w_{i}{\left\|y_{\text{seg},N_{\text{seg}}-i+1}\left(\alpha \right)-Cz_{\text{sig},m+i-1}-h\right\|}_{2}^{2} & \text{left direction} \end{array}$$

where *C* is the rotation matrix, *h* ∈ *R*^3^ is the magnetic disturbance vector and *w_i_* is a positive weighting factor.

If the segment location (the leftmost position of the segment) is the *m*-th cell, *f*(*m, α*, direction) is small assuming *α* is the correct walking parameter. Thus the location estimation problem can be solved by minimizing *f*(*m, α*, direction) over 1 ≤ *m* ≤ *N_cell_* − *N_seg_* + 1. This approach sometimes could give a wrong answer since *f*(*m,α*, direction) could be small even if the segment location is not the *m*-th cell. As a fail-safe measure, we check whether the computed *C* in Equation (3) satisfies the assumption that the attitude change is small in Equation (1), and the process is given in Equations (4) – (7).

Suppose a person is standing before starting to walk. Let *y_a,init_* ∈ *R*^3^ be the accelerometer output of the sensor attached to the body. Then, there is no external acceleration since there is no movement. Thus, *y_a,init_* only contains the gravitational acceleration:

(4) $$y_{a,\text{init}}\approx C\left[\begin{array}{c} 0\\ 0\\ g_{\text{earth}} \end{array}\right]$$

where *g_earth_* is the gravitational acceleration.

Let *b_z_* be the unit vector in the direction of *y_a,init_*:

$$b_{z}=\frac{y_{a,\text{init}}}{\left\|y_{a,\text{init}}\right\|}$$

Note that *b_z_* is computed from *y_a,init_*, which can be obtained if a person stands for 1 to 2 seconds before starting to walk. For example, consider the body coordinate system in Figure 3. In this case, *b_z_* becomes the *x* axis unit vector of the body coordinate system. During walking, the *b_z_* direction maintains the upward direction. Thus the following can be assumed:

(5) $$b_{z}\approx C\left[\begin{array}{c} 0\\ 0\\ 1 \end{array}\right]$$

where *C* is the rotation matrix from Equation (3).

The similarity of two unit vectors in Equation (5) is tested by measuring the angle between them. The angle *θ* between the vectors *a* ∈ *R*^3^ and *b* ∈ *R*^3^ can be computed by

(6) $$\theta =\cos^{-1}\left(\alpha^{\prime }b\right)$$

Thus the two vectors are similar if *a*′*b* is large (that is, if *θ* is small).

Using Equation (6), we introduce *f̄*(*m, α*)

(7) $$\bar{f}\left(m,\alpha ,\text{direction}\right)=\{\begin{array}{ll} f(m,\alpha ,\text{direction}), & \text{if}b_{z}^{'}C{\left[\begin{array}{ccc} 0 & 0 & 1 \end{array}\right]}^{'}>\gamma_{1}\\ \infty , & \text{otherwise} \end{array}$$

where *γ*_1_ is a positive threshold value.

Now the segment location can be estimated by minimizing *f̄*(*m, α*, direction). We note that the minimizing *m* still may not be the correct position although it rarely happens. We introduce a quantitative index *g*(*α*, direction) representing the confidence of the estimated location by minimizing Equation (7). An index *g*(*α*, direction) is defined by

(8) $$g\left(\alpha ,\text{direction}\right)=\alpha_{1}\exp\left(b_{1}{\bar{f}}_{1}\left(\alpha ,\text{direction}\right)\right)+\alpha_{2}\exp\left(b_{2}\frac{{\bar{f}}_{1}\left(\alpha ,\text{direction}\right)}{{\bar{f}}_{2}\left(\alpha ,\text{direction}\right)}\right)$$

where *f̄*_1_(*α*, direction) and *f̄*_2_(*α*, direction) are the minimum and second minimum values of *f̄*(*m, α*, direction) for 1 ≤ *m* ≤ *N_cell_* − *N_seg_* + 1. The coefficients *a*_1_*, b*_1_*, a*_2_ and *b*_2_ are all positive. Note that *g*(*α*, direction) is small if the absolute and relative (with respect to the second minimum value) minimum values are small. In this case, it is more likely that the minimizing *m* is the true cell position. *g*(*α*, direction) plays a similar role as the estimation error covariance of the estimated cell position and *g* is called a pseudo error covariance. In the minimization process, we also compute *C* and *h* in Equation (3). If *g*(*α*, direction) is small, the computed *C* and *h* are generally more accurate.

In the minimization of *f̄*, we have to find *C* (rotation matrix) and *h* in Equation (3). This minimization problem is often encountered in computer vision problems. There is an analytic solution to compute *C* and *h* and the result [20] is given in Lemma 1.

**Lemma 1** *Let a_i_* ∈ *R*^3^ *(*1 ≤ *i* ≤ *n) and b_i_* ∈ *R*^3^ *be corresponding vectors in R*^3^. *Let w_i_* ∈ *R*^3^ *be a positive weighting factor*.

*The rotation matrix C and a translation vector h* ∈ *R*^3^ *satisfying*

(9) $$\underset{C,h}{\min}\sum_{i=1}^{n}w_{i}{\left\|a_{i}-Cb_{i}-h\right\|}_{2}^{2}$$

*are given by*

$$C=V\left[\begin{array}{ccc} 1 & 0 & 0\\ 0 & 1 & 0\\ 0 & 0 & \det\left(VU^{'}\right) \end{array}\right]U^{'},h=ā-C\bar{b}$$

*where*

$$ā=\frac{\sum_{i=1}^{n}w_{i}a_{i}}{\sum_{i=1}^{n}w_{i}},\bar{b}=\frac{\sum_{i=1}^{n}w_{i}b_{i}}{\sum_{i=1}^{n}w_{i}},{ã}_{i}=a_{i}-ā,{\tilde{b}}_{i}=b_{i}-\bar{b},S=\sum_{i=1}^{n}w_{i}{ã}_{i},{\tilde{b}}_{i}^{'}$$

*Orthogonal matrices U and V are from the singular value decomposition of S*:

$$S=U\text{Σ}V^{'}$$

*where* Σ *is a diagonal matrix*.

Using Lemma 1, we can compute *C* and *h* in Equation (3) easily, and instead of using a numerical optimization, *C* and *h* are given in the closed-form. The singular value decomposition of a 3 × 3 matrix, which is easy to compute, is the most computationally demanding part. Thus the computational cost of finding *C* and *h* in Equation (3) is small. This is important since Equation (3) is used as an inner algorithm in Equations (3) and (14) thus is computed many times.

In Equation (9), *n* should be at least 2. Otherwise, the problem becomes underdetermined and there are infinitely many solutions. Thus *N_seg_* in Equation (3) should be at least 2. This number is the minimum when there is no magnetic noise (sensor noise and temporary magnetic disturbances). *N_seg_* should be much larger (practically, at least 5) to obtain a reliable solution.

## 4. Segment Data Generation

In this section, the generation of segment data *y_seg,i_* (used in Section 3) is explained.

**Figure 5.**
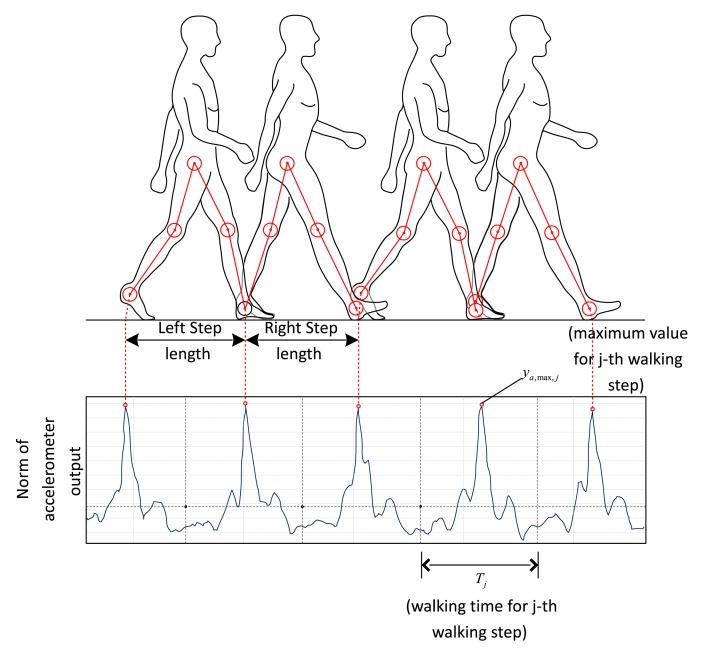
Norm of accelerometer output during walking.

In Figure 5, a typical plot of the norm of the accelerometer output during walking is given. It is not difficult to identify each walking step from the norm data. Let *y_a,max,j_* be the maximum norm value of the j-th walking step and *T_j_* be the walking time. It is known that the walking length of the *j*-th step is proportional to *y_a,max,j_* [8]. Thus walking length *l_j_* for each step can be estimated using the following formula:

(10) $$l_{j}=\alpha y_{a,\max,j}$$

where *α* is a person-dependent parameter satisfying the following:

(11) $$\alpha_{\min}\leq \alpha \leq \alpha_{\max}$$

where *α*_min_ and *α*_max_ can be determined from experiments on a number of people.

Equation (10) is not a new result and there are many similar algorithms [21]. Since the walking distance estimation algorithm is not the main topic of this paper, any other algorithms can be used.

A walking segment consists of several walking steps. Whenever the walking direction change is larger than a certain threshold value, a new segment is formed. Thus a segment consists of almost straight line walking data. To prevent the segment length from becoming too long, a new segment is formed if the segment length is larger than a certain threshold value. A new segment is formed if the following are not satisfied (*γ*_2_ and *γ*_3_ are positive parameters):

- Small direction change
  (12) $$\left\|y_{m,i}-y_{m,j}\right\|<\gamma_{2}$$
- Segment not too long
  (13) $$\sum y_{a,\max,j}<\gamma_{3}$$

The segment index is denoted by *k* and the number of walking steps in the *k*-th segment is denoted by *M_k_*. With *j* denoting the walking step index, the walking step interval *k*_1_ ≤ *j* ≤ *k_M_k__* is assumed to belong to the *k*-th segment.

**Figure 6.**
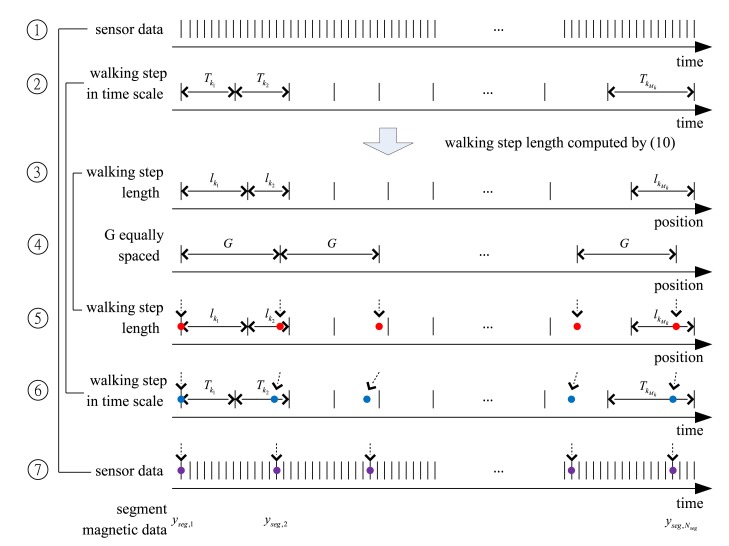
*y_seg,i_* generation: (1,7), (2,6) and (3,5) are the same graph drawn twice for the explanation.

Note that *y_seg,i_* and *y_seg,i_*_+__1_ are two magnetic data obtained at positions with distance *G*. Using the computed *l_j_, y_seg,i_* data is constructed as follows (see Figure 6).

- Steps 1–3: Using accelerometer data, walking step length *l_j_* is computed for each walking step *j* using Equation (10).
- Steps 4–5: Based on the computed *l_j_*, the walking path is divided into positions with equal spacing (by length *G*).
- Steps 6–7: Using a simple interpolation algorithm, the time instances corresponding to these equally spaced positions are computed. The magnetic sensor data at these time instances are denoted by *y_seg,i_*.

## 5. Proposed Location Estimation Algorithm

In this section, a location estimation algorithm using the results in Sections 3 and 4 is provided. Let *m̂_k_, α̂_k_* and *ĥ_k_* be the estimated values of *m, α* and *h* after the *k*-th segment data is processed. Let *P_k_* ∈ *R* be a pseudo estimation error covariance (see the discussion for Equation (8)) after the *k*-th segment data is processed. A small *P_k_* value means that *m̂_k_* is more accurate.

### 5.1. Initialization

When a sensor is installed on a person for the first time, we do not have any information on *α* and *h*. If *h* is unknown, it is not possible to determine the walking direction. In this case, initial values of *α* and *h* should be found in addition to the location *m*. The location and initial value estimation problem can be formulated as a minimization problem over all possible combinations of parameters:

(14) $${\bar{f}}^{*}=\underset{\text{right},\text{left direction}}{\min}\left[\underset{\alpha_{\min}\leq \alpha \leq \alpha_{\max}}{\min}\left(\underset{1\leq m\leq N_{\text{cell}}-N_{\text{seg}+1}}{\min}\bar{f}\left(m,\alpha ,\text{direction}\right)\right)\right]$$

where f̄* is the minimum value. Let m*, h*, C* and α* be the minimizing parameters. The direction (right or left) and *m* are discrete parameters. The continuous parameter *α* can be discretized in the minimization problem with the discretization interval Δ*α*: that is, discrete values *α*_min_*, α*_min_ + Δ*α, α*_min_ + 2Δ*α*, ⋯, *α*_max_ are used in the minimization of Equation (14).

In the minimization problem, the direction is determined and the initial values of *α* and *h* are given. If the value *g* (see Equation (8)) is larger than a threshold value *γ*_4_, the result is not used. We then proceed to the next segment to determine the initial values.

Suppose the minimization problem is solved for the first walking segment. Then *α̂*_1_, *ĥ*_1_ and *P*_1_ are given by

$${\hat{\alpha }}_{1}=\alpha^{*},{ĥ}_{1}=h^{*},P_{1}=g\left(\alpha^{*},{\text{direction}}^{*}\right)$$

where direction* represents the determined direction (right or left). Symbols *α̂_k_* and *ĥ_k_* denote the estimated values of *α* and *h* using the segment data up to the *k*-th segment.

### 5.2. Walking Direction Determination after the Initialization

Suppose we have segment magnetic data *y_seg,i_* (1 ≤ *i* ≤ *N_seg_*) for a new segment. From Equation (1), we know that (assuming *y_seg,i_* is measured on the *m* + *i* − 1-th cell)

(15) $$y_{\text{seg},i}=Cz_{\text{sig},m+i-1}+h$$

where *C, h* and *m* are unknown.

In the initialization step, we obtained *ĥ*_1_ = h* and *Ĉ*_1_ = C*. We can assume that *h* does not change much during walking. The rotation matrix *C* could take two values. If the direction of the current segment is the same as that of the first segment, the rotation matrix *C* is given by

(16) $$C\approx {Ĉ}_{1}$$

If the direction is opposite, the rotation matrix *C* can be obtained by rotating the *z* axis of the body coordinate system in the first segment by *π* rad along the *b_z_* axis (since a person has turned *π* rad along this axis):

(17) $$C\approx C\left(b_{z},\pi \right){Ĉ}_{1}$$

where *C*(*b_z_, π*) is the rotation matrix representing a *π* rad rotation along the *b_z_* axis [22].

Thus from Equations (15) – (17), we have

(18) $$y_{\text{seg},i}\approx \{\begin{array}{ll} {Ĉ}_{1}z_{\text{sig},m+i-1}+{ĥ}_{1} & \text{if the same direction}\\ C\left(b_{z},\pi \right){Ĉ}_{1}z_{\text{sig},m+i-1}+{ĥ}_{1} & \text{if the opposite direction} \end{array}$$

Since *m* is unknown, we cannot use *z_sig,m_*_+_*_i_*_−1_ in Equation (18). Instead, the mean value of *z_sig,i_* is used. Let *z_sig,mean_* and *y_seg,mean_* be defined by

$$z_{\text{sig},\text{mean}}=\frac{1}{N_{\text{cell}}}\sum_{m=1}^{N_{\text{cell}}}z_{\text{sig},m},y_{\text{seg},\text{mean}}=\frac{1}{N_{\text{seg}}}\sum_{i=1}^{N_{\text{seg}}}y_{\text{seg},i}$$

Based on the preceding observation, the direction determination algorithm is summarized as follows:

- The direction is determined as the same direction with the first segment if the following is satisfied
  (19) $$\left\|y_{\text{seg},\text{mean}}-{Ĉ}_{1}z_{\text{mean}}+{ĥ}_{1}\right\|\leq \min\left(\gamma_{5},\left\|y_{\text{seg},\text{mean}}-C\left(b_{z},\pi \right){Ĉ}_{1}z_{\text{mean}}+{ĥ}_{1}\right\|\right)$$
  where *γ*_5_ is a positive parameter.
- The direction is determined as the opposite direction to the first segment if the following is satisfied
  (20) $$\left\|y_{\text{seg},\text{mean}}-C\left(b_{z},\pi \right){Ĉ}_{1}z_{\text{mean}}+{ĥ}_{1}\right\|\leq \min\left(\gamma_{5},\left\|y_{\text{seg},\text{mean}}-{Ĉ}_{1}z_{\text{mean}}+{ĥ}_{1}\right\|\right)$$
- The direction is undetermined if neither Equation (19) nor Equation (20) is satisfied. In this case, the method in the initialization step should be used.

We note that *ĥ_k_*_−1_ and *Ĉ_k_*_−1_ (which are generally more accurate) could be used for the *k*-th segment instead of *ĥ*_1_ and *Ĉ*_1_.

### 5.3. Location Estimation Algorithm for k-th Walking Segment

Suppose the location is estimated up to the *k* − 1-th segment and we have computed *α̂_k_*_−1_*, ĥ_k_*_−1_ and *P_k_*_−1_. The location estimation algorithm for the *k*-th walking segment is explained.

- The direction is estimated using the method in Section 5.2.
- The position (*m*) and *α* are estimated using the following optimization problem:
  (21) $${\bar{f}}^{*}=\underset{\alpha_{\min}\leq \alpha \leq \alpha_{\max}}{\min}\left(\underset{1\leq m\leq N_{\text{cell}}-N_{\text{seg}}+1}{\min}\bar{f}\left(m,\alpha ,\text{direction}\right)\right)$$
- Let $\alpha_{k}^{*}$, $h_{k}^{*}$, $C_{k}^{*}$ and $m_{k}^{*}$ be the minimizing solution and $g_{k}^{*}$ be the *g* value at the minimizing solution.
- *α̂_k_*, *ĥ_k_*, *Ĉ_k_* and *P_k_* are updated as follows:
  (22) $$\begin{array}{ll} {\hat{\alpha }}_{k} & =\frac{g_{k}^{*}}{P_{k-1}+g_{k}^{*}}{\hat{\alpha }}_{k-1}+\frac{P_{k-1}}{P_{k-1}+g_{k}^{*}}\alpha_{k}^{*}\\ {ĥ}_{k} & =\frac{g_{k}^{*}}{P_{k-1}+g_{k}^{*}}{ĥ}_{k-1}+\frac{P_{k-1}}{P_{k-1}+g_{k}^{*}}h_{k}^{*}\\ {Ĉ}_{k} & =C_{k}^{*}\\ P_{k} & =\frac{P_{k-1}g_{k}^{*}}{P_{k-1}+g_{k}^{*}} \end{array}$$

In Equation (22), *α̂_k_*_−1_ is the estimated value of *α* based on up to the *k* − 1-th segment while $\alpha_{k}^{*}$ is the estimated value of *α* based on the *k*-th segment. If *P_k_*_−1_ and $g_{k}^{*}$ are the estimation error covariances of *α̂_k_*_−1_ and ${\hat{\alpha }}_{k}^{*}$, then *α̂_k_* in Equation (22) is the optimal estimate and *P_k_* becomes the estimation error covariance of *α̂_k_* [23]. Similar logic applies to *ĥ_k_*. Since *P_k_*_−1_ and ${ĝ}_{k}^{*}$ are not the covariances, the computed *α̂_k_* is just a suboptimal estimate (lacking theoretical justification) and *P_k_* is not the estimation error covariance.

Some comments are given for the computational aspects of Equation (14). The computation of *f̄* depends on *c*_1_*N_seg_* + *c*_2_, where *c*_1_ and *c*_2_ are constants. To solve Equation (21), *f̄* should be computed for all possible combinations. Thus the computation of Equation (21) depends on

(23) $$\left(\frac{\alpha_{\max}-\alpha_{\min}}{\Delta \alpha }+1\right)\times \left(N_{\text{cell}}-N_{\text{seg}}+1\right)\times \left(c_{1}N_{\text{seg}}+c_{2}\right)$$

The largest number in Equation (23) is *N_gird_*, which is proportional to 1/*G*. Thus the computational cost is inversely proportional to the cell size *G*.

## 6. Experiments

**Figure 7.**
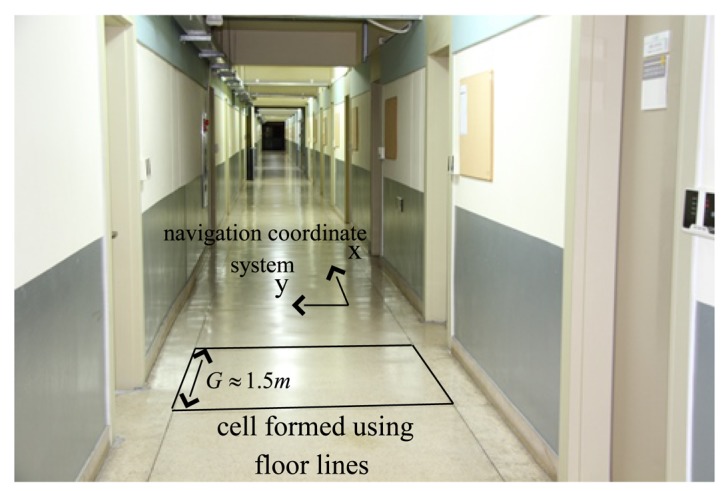
Cells using floor lines in the corridor.

Experiments were done on the 4th floor of building 7 in the University of Ulsan. In the corridor, there are floor lines forming cells with approximately the same size (see Figure 7). There are a total of 60 cells (*N_cell_* = 60) and the total length is 89.7 m (the average length of each cell is 1.495 m).

**Figure 8.**
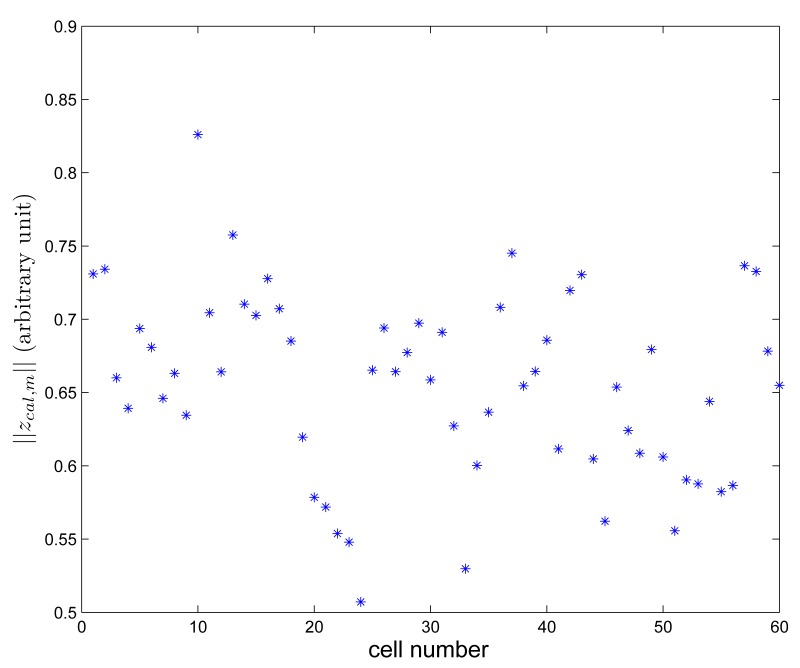
‖*z_sig,m_*‖ plot.

Magnetic signature data *z_sig,m_* is recorded using the procedure in Section 2. The norm of *z_sig,m_* is given in Figure 8. It can be seen that there are significant location-to-location variations.

**Figure 9.**
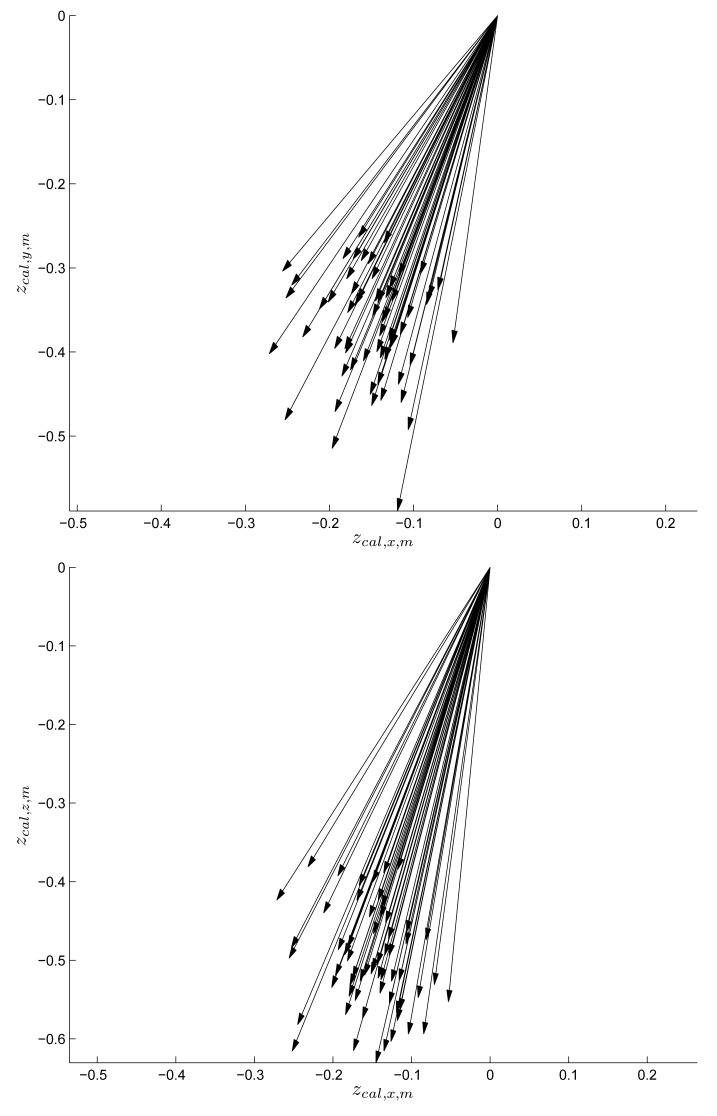
Vector plot of *z_sig,m_*.

To see the magnetic signature variation in the vector form, *x* and *y* elements of *z_cal,m_* are given in the left graph of Figure 9 and *x* and *z* elements of *z_cal,m_* are given in the right graph. Each vector represents the vector element of *z_cal,m_* in a cell, where location-dependent magnetic field differences can be noticed. We can also observe that the magnetic disturbance from the building is small compared with the earth's magnetic field. Otherwise, the vector distribution would be much wider.

**Figure 10.**
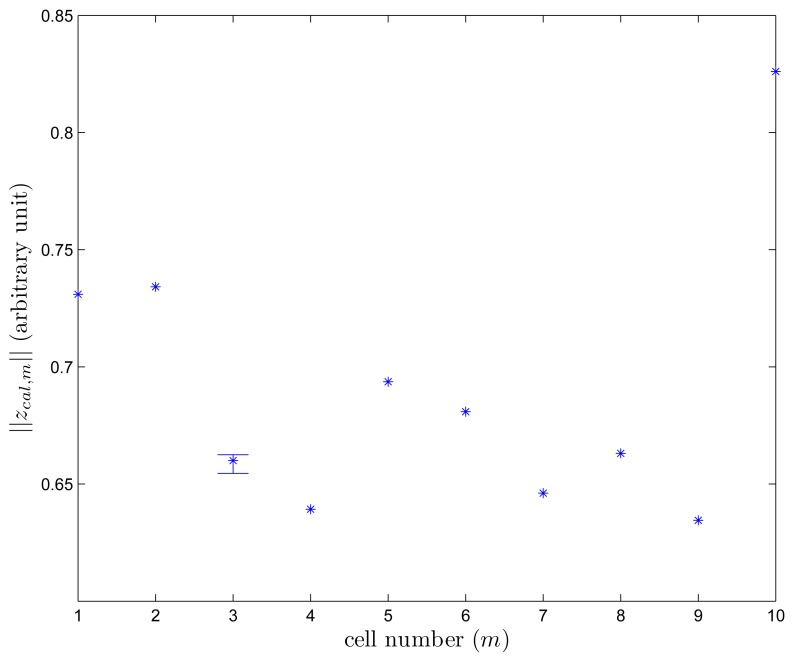
‖z*_sig,m_*‖ plot for 1 ≤ *m* ≤ 10 with time-dependent variation of ‖*z_cal_*_,3_‖.

To use this location-dependent variation in *z_sig,m_* as a magnetic signature, the value should be fairly time-invariant. To investigate the time dependence of *z_cal,m_*, we recorded *z_sig,m_* (for *m* = 3) for seven days (six times a day at 10 am, 12 pm, 2pm, 6 pm, 8 pm, and 10 pm). In Figure 10, we can see that the time-dependent variation is small compared with the location-dependent variation: 0.6546 ≤ *z_sim,_*_3_ ≤ 0.6625. Since we use multiple cell data (segment data) to estimate the location, we believe this small time-dependent variation can be ignored. We conjecture this variation is due to sensor noise and changes of the environment (door opening/closing, people passing).

In the first walking experiment, a person walked from the end of cell 1 to the end of cell 60, a length of 89.7 m. This walking test was performed by 9 people whose information is given in Table 1. The parameters of the proposed algorithm are given in Table 2.

The results are given in Table 3. The walking paths are divided into 4 to 5 segments according to the rules in Section 4. The second column (*m̂*) represents the estimated location (in cell numbers) for each segment. For example, the estimated locations are 1 → 15 → 29 → 43 in the case of person (A). If a segment is too short, it is not used for the location estimation. In this case, the symbol ‘?’ is used. This can happen in the final segment as in the cases of person (B), (C), (D) and (G). The third column represents the walking distance estimated by the proposed algorithm and the fourth column represents the percentage error in the estimated walking distance. The absolute average error percentage is 3.1%, which is rather good considering the fact that the walking parameter *α* is automatically estimated.

The fifth and sixth columns indicate *α̂_k_* and *ĥ_k_* (for the final segment). The *α̂_k_* values are person-dependent. We can observe that women have smaller *α̂_k_* values presumably because they have smaller walking step lengths.

Persons (B) – (H) used the same belt (a military-style belt with some metal parts) where the sensor is attached. In the case of persons (A) and (I), the sensor is inserted inside the trousers (around the belt position). It can be seen that *ĥ* values are similar for persons (B)–(H), which is probably due to the fact *h* is affected by the same metal parts in the belt.

**Figure 11.**
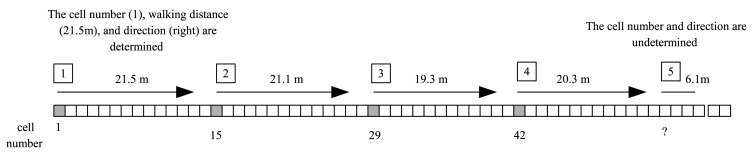
Estimated location in Table 4 (first 5 segments).

**Figure 12.**
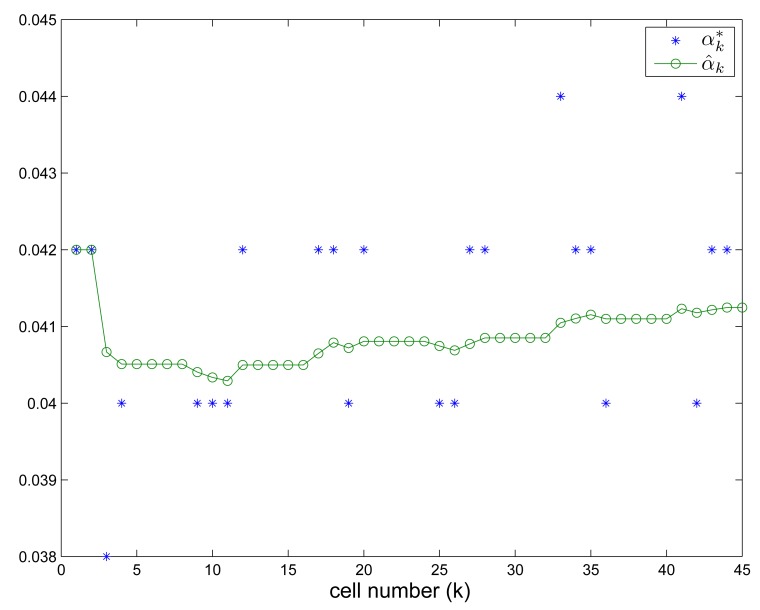
$\alpha_{k}^{*}$ and ${\hat{\alpha }}_{k}^{*}$.

In the second walking experiment, a person walked three round trips between cell 1 and cell 60. The estimated location result is given in Table 4. In Figure 11, the estimation results for the first five segments are illustrated with four segments in the first row and one segment in the second row in Table 4. As in Table 3, ‘?’ is used to indicate that the location is not estimated since the segment length is too short. In the second row, it can be seen that a person walked to the right 1 → 15 → 29 → 42 (cell numbers). When a person turned around at the end of cell 60, short segments are formed due to the large direction change (see the segment generation rule in Section 4). The location estimation is not used for these short segments. After turning around, when a person walked to the left (4th row), the proposed algorithm identifies the direction change correctly. In the second experiment, it can be seen that the walking direction can be correctly identified in all segments. The total walking length is 538.2 m (89.7 m × 6) and the sum of the estimated walking length of all segments is 538.2m (two values were accidentally the same). The same experiment was also done for person (B) and the estimated distance is 536.8 m. To see how *α̂_k_* changes during walking, ${\hat{\alpha }}_{k}^{*}$ and *α̂_k_* values for person (A) are given in Figure 12. Through this long distance walking test, we can verify that the proposed algorithm can estimate the direction, location and walking distance relatively accurately.

To test the location estimation accuracy, persons (A) and (B) walked on four paths as shown in Table 5. One segment is formed for each path by setting *γ*_3_ to a large value. When a person starts from the center of cell 30, the location is correctly estimated as cell 30 in all cases. When a person starts from the borderline of cells 9 and 10, the location is estimated either as cell 9 or 10. In all cases, the location is correctly estimated with one cell length resolution. The estimated walking distance is also given in Table 5, where the true distance is 29.3 m.

**Figure 13.**
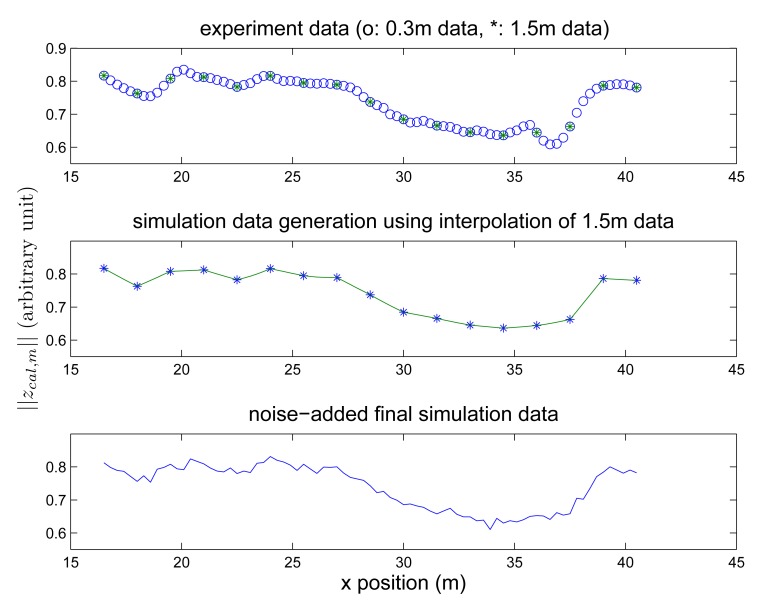
Simulation data generation example: (top) ‖*z_sig,m_*‖ with 0.3 m cell size experiment data, (middle) simulation data generation using interpolation of 1.5 m data (bottom) noise-added final simulation data.

Finally, we investigate how *G* (cell size) and *N_seg_* (number of magnetic data in a segment) affect the location estimation performance. In the experiment, the cell size was 1.5 m and the magnetic signature data was obtained at positions 1.5 m apart. In Figure 13 (left figure), the magnetic signature data was obtained with fine cells (0.3m cell size) along 24 m intervals. There is no significant fluctuation in magnetic signature data between 1.5 m cell magnetic signature data (marked with ‘*’ in Figure 13). Based on this observation, simulated magnetic signature data is generated assuming that the cell size is 0.1 cm in Figure 13 (right figure): magnetic signature data (cell size 0.1 cm) is interpolated (a linear interpolation) using 1.5 m cell size magnetic signature data (middle graph in Figure 13) and noise is added to the interpolated data (bottom graph in Figure 13). Using this simulated data, we investigated the effect of *G* and *N_seg_*. The simulation data in Figure 13 is just a part of the simulation data to show how the simulation data is generated. Although there is no large magnetic signature variation between 1.5 m magnetic data, there could be large variation if there are magnetic anomalies such as fire extinguishers between the measurement points.

To focus on the effect of *G* and *N_seg_*, we assume that *α, h* and *C* are exactly known. The *y_seg,i_* data is generated by adding noise to simulated magnetic signature data *z_sig,m_*. When the noise is too small, the location estimation performance is good for all *G* and *N_seg_* values. Thus large noise is used to generate *y_seg,i_* (magnetic data obtained during walking).

Three different cell sizes (0.5 m, 1.0 m, 1.5 m) and different *N_seg_* (1 ≤ *N_seg_* ≤ 10) are used in the simulation. Assuming that the total length of a corridor is 88.5 m, we checked whether the algorithm correctly identifies the location for every location. For example, if *G* = 1.5 *m* and *N_seg_* = 2, then the total number of cells is *N_cell_* = 60. Assuming that the starting *x* position is 0 m, we checked whether the algorithm gives the correct location. Then assuming that the starting *x* position is 1.5 m, we checked again. This process is repeated until the starting *x* position is 87 m (so that the final position of the segment is 88.5 m). If the estimated location error is smaller than 1m, we consider the location estimation successful. We compute the success rate for the given combination (*G* and *N_seg_*) as the average value of 100 simulation results.

The simulation result is given in Table 6. We can see that the success rate increases with *N_seg_*. This is not surprising since large *N_seg_* means that more magnetic data is used for location estimation and thus the location estimation is robust to sensor noise. However, large *N_seg_* means large latency (for example, *G* = 1.5 and *N_seg_* = 7 mean that we should walk (*N_seg_* − 1)*G* = 9 m to estimate the location).

Now the effect of *G* on the location estimation performance is investigated. There are drawbacks and merits of using a smaller cell size. For the same *N_seg_* value, a larger *G* value gives a better success rate. For example, for *N_seg_* = 5, the success rate is 69.08 % for a 0.5 m cell. The success rate 91.80% for a 1.5 m cell is significantly better. Also, a smaller cell requires more computation: *N_cell_* is proportional to 1/*G* and thus the computational complexity is proportional to 1/*G*. On the other hand, the merit of a smaller cell size is its small latency. If we compare the cases of (*N_seg_, G*) = (10, 0.5) and (*N_seg_, G*) = (7, 1.0), the success rate is similar. However, the required walking length for *G* = 0.5 is 4.5 m while the required walking length for *G* = 1 is 6 m. Thus, we can estimate location with a smaller walking length if we use a smaller cell size.

One guideline for *N_seg_* and *G* is given as follows: *G* should be chosen based on the required estimation accuracy. If 1.5 m estimation error is the acceptable upper limit, *G* =1.5 m can be chosen. Next, *N_seg_* can be chosen based on the magnetic disturbance level (from on-off electric machines for example). The larger *N_seg_* chosen, the more robust the estimated location becomes at the sacrifice of large latency.

## 7. Conclusions

In this paper, the location in the one-dimensional corridor is estimated using location-dependent ambient magnetic fields inside buildings. The sensor (inertial/magnetic) is attached to the belt. The walking length is estimated using the accelerometer output. The magnetic data during walking is compared with pre-recorded magnetic signature data.

The main contribution is that person-dependent parameters (walking parameter ***α*** and hard iron parameter *h*) are adaptively estimated in the proposed algorithm. Thus no person-dependent calibration procedure is required and it can be used for the location estimation as soon as the device is attached to the belt.

The proposed algorithm was tested on 9 people (weight: 48–72 Kg, height: 155–181 cm, age: 22–47). With 89.7 m walking tests, the average distance estimation error was 3.1%. The location estimation was also accurate with the resolution depending on the cell size (about 1.5 m).

The proposed algorithm is not a full-scale localization algorithm such as in [24]. It essentially solves the location estimation problem of a one segment. There is no dynamic modeling about people or map matching algorithms. The proposed algorithm can be used in a full-scale localization problem as a useful subroutine. For example, the measurement equations of filters in [10,11] could be replaced by the proposed algorithm.

Future work is to increase the location estimation accuracy and to extend the result to two-dimensional location estimation problem.

## Figures and Tables

**Table 1.** Information about people in the first walking experiment.

**Person**	**Age**	**Weight (Kg)**	**Height (Cm)**	**Sex**
(A)	47	67	179	male
(B)	29	68	169	male
(C)	28	72	181	male
(D)	24	64	163	male
(E)	27	61	176	male
(F)	28	62	169	male
(G)	23	48	158	female
(H)	22	55	172	female
(I)	25	60	155	female

**Table 2.** Parameters used in the experiment.

**Parameter**	**Values**	**Related Equation**
*γ*_1_	0.8	Equation (7)
*γ*_2_	0.2	Equation (12)
*γ*_3_	$\frac{15}{\alpha_{\min}}$	Equation (13)
*γ*_4_	1.5	Section 5.1
*a*_1_*, a*_2_*, b*_1_*, b*_2_	0.5,0.5,1,1	Equation (8)
*α*_min_, *α*_max_, Δ*α*	0.03, 0.05, 0.002	Equations (11) and (14)

**Table 3.** 60 cell (89.7 m) walking test (‘?’ symbol means the segment is too short for the location estimation).

**Person**	***m̂***	**Estimated Distance (m)**	**Error (%)**	***α̂***	*h****^***’
(A)	1,15,29,43	91.3	1.8	0.042	[0.12 −0.14 0.06]
(B)	1, 14,28,42,(?)	88.4	−1.4	0.042	[−0.37 0.47 −0.33]
(C)	1, 17, 34, (?)	84.0	−6.3	0.047	[−0.30 0.48 −0.38]
(D)	1, 15,29,44,(?)	85.4	−4.8	0.042	[−0.34 0.44 −0.39]
(E)	1,17,33,49	85.1	−5.1	0.047	[−0.28 0.50 −0.44]
(F)	1,17,32,47	91.6	2.1	0.046	[−0.26 0.59 −0.38]
(G)	1, 15,29,42,(?)	90.3	0.7	0.040	[−0.26 0.45 −0.72]
(H)	1,13,25,37,50	85.2	−5.1	0.035	[−0.21 0.49 −0.67]
(I)	1,15,28,42	88.3	−1.0	0.042	[0.20 −0.12 0.09]
		absolute mean error	3.1		

**Table 4.** Long walking test: three round trips between cell 1 and cell 60 (‘?’ symbol means the segment is too short for the location estimation).

**Action**	**Estimated Cell Number (Estimated Walking Distance)**	**Estimated Direction**
Walking to the right direction	1(21.5), 15(21.1), 29(19.3), 42(20.3)	right
Turning around	?(6.1),?(0.5),?(3.9),?(0.7)	
Walking to the left direction	57(20.7), 43(20.2), 29(20.6), 16(21.5)	left
Turning around	?(0.6), ?(0.5), ?(0.5), ?(0.6)	
Walking to the right direction	1(21.1), 15(21.4), 29(20.7), 42(21.4)	right
Turning around	?(4.5), ?(0.5), ?(0.5), ?(0.6)	
Walking to the left direction	59(20.1), 46(20.5), 32(21.2), 19(21.7)	left
Turning around	?(5.3), ?(0.5), ?(0.5), ?(0.6)	
Walking to the right direction	1(22.2), 15(21.6), 29(21.2), 43(20.6)	right
Turning around	?(4.5), ?(0.6), ?(0.6), ?(0.6)	
Walking to the left direction	60(22.4), 45(20.5), 32(21.4), 18(21.6), ?(4.8)	left

**Table 5.** Location estimation accuracy test.

**Path**	**Person (A) Estimated Cell Number (Estimated Walking Distance)**	**Person (B) Estimated Cell Number (Estimated Walking Distance)**
Borderline between cell 9 and 10	10 (29.3)	9(31.5)
→ center of cell 30		

Center of cell 30→	30 (30.2)	30 (30.9)
borderline between cell 50 and 51		

Borderline between cell 50 and 51	51 (31.9)	51 (32.9)
→ center of cell 30		

Center of cell 30→	30 (32.2)	30 (30.8)
borderline between cell 9 and 10		

**Table 6.** Location estimation performance with different *N_seg_* and cell sizes (100 simulations, estimation error smaller than 1m is considered as success).

	**Location Estimation Success Rate (%)**		

***N****_seg_*	**0.5 m Cell**	**1.0 m Cell**	**1.5 m Cell**
1	28.90	33.10	40.59
2	39.90	47.75	60.29
3	51.02	60.65	74.42
4	61.32	71.40	85.46
5	69.08	80.39	91.80
6	76.39	87.33	95.74
7	82.12	92.18	97.91
8	86.65	95.06	99.00
9	89.96	96.87	99.57
10	92.87	98.14	99.76
